# Clinical Efficacy and Safety of a Novel Antifungal, Fosmanogepix, in Patients with Candidemia Caused by Candida auris*:* Results from a Phase 2 Trial

**DOI:** 10.1128/aac.01419-22

**Published:** 2023-04-06

**Authors:** Jose A. Vazquez, Peter G. Pappas, Kenneth Boffard, Fathima Paruk, Paul A. Bien, Margaret Tawadrous, Eric Ople, Pamela Wedel, Iwona Oborska, Michael R. Hodges

**Affiliations:** a Medical College of Georgia, Augusta University, Augusta, Georgia, USA; b University of Alabama at Birmingham, Birmingham, Alabama, USA; c Milpark Hospital, University of the Witwatersrand, Johannesburg, South Africa; d Steve Biko Academic Hospital, University of Pretoria, Pretoria, South Africa; e Amplyx Pharmaceuticals Inc., San Diego, California, USA; f Pfizer Inc., Groton, Connecticut, USA

**Keywords:** intensive care unit, APX001, *Candida auris*, Gwt1 inhibitor, candidemia, fosmanogepix

## Abstract

Fosmanogepix (FMGX), a novel antifungal available in intravenous (IV) and oral formulations, has broad-spectrum activity against pathogenic yeasts and molds, including fungi resistant to standard of care antifungals. This multicenter, open-label, single-arm study evaluated FMGX safety and efficacy for treatment of candidemia and/or invasive candidiasis caused by Candida auris. Eligible participants were ≥18 years, with established candidemia and/or invasive candidiasis caused by C. auris, (cultured within 120 h [for candidemia] or 168 h [for invasive candidiasis without candidemia] with accompanying clinical signs) and limited treatment options. Participants were treated with FMGX (≤42 days; loading dose: 1000 mg IV twice daily [Day 1], followed by 600 mg IV once daily [QD]). Switching to oral FMGX 800 mg QD was permitted from Day 4. Primary endpoint was treatment success (survival and clearance of C. auris from blood/tissue cultures without additional antifungals) at the end of the study treatment (EOST), assessed by an independent data review committee (DRC). Day 30 survival was a secondary endpoint. *In vitro* susceptibility of *Candida* isolates was assessed. Nine participants with candidemia (male:6, female:3; 21 to 76 years) in intensive care units in South Africa were enrolled; all received IV FMGX only. DRC-assessed treatment success at EOST and Day 30 survival were 89% (8/9). No treatment related adverse events or study drug discontinuations were reported. FMGX demonstrated potent *in vitro* activity against all C. auris isolates (MIC range: 0.008 to 0.015 μg/mL [CLSI]; 0.004–0.03 μg/mL [EUCAST]), with the lowest MICs compared to other antifungals tested. Thus, the results showed that FMGX was safe, well-tolerated, and efficacious in participants with candidemia caused by C. auris.

## INTRODUCTION

Candida auris is a fungal pathogen that was first identified in 2009 and has since emerged as a global threat. C. auris isolates have been implicated in nosocomial outbreaks worldwide, with 5 distinct clades classified by region of independent emergence. Based on current profiling, these clades are referred to as Clade I (South Asian), Clade II (East Asian), Clade III (African), Clade IV (South American), and Clade V (Iranian) with different genetic determinants of resistance and resulting antifungal resistance profiles. Despite the classification of each clade based on the initial geographical region of detection, transmission in other areas has been reported, with multiple clades identified in Canada, Kenya, and the United States (1–7).

Invasive C. auris infections are associated with high mortality rates, with 60% of hospitalizations due to C. auris infections resulting in death (8). These rates are higher than the observed mortality rates for candidemia/invasive candidiasis, which are estimated to be 25% overall and 31%, respectively, for patients ≥65 years of age (9).

C. auris is well known for its multi-drug resistant characteristics, with most isolates resistant to fluconazole, an important first-line antifungal agent (3, 6, 10, 11). Furthermore, 41% of C. auris isolates from a global study were found to be resistant to 2 or more classes of antifungals (8). Indeed, echinocandins are now generally recognized as the drugs of choice for the treatment of C. auris infections (12). Increased rates of resistance, especially to the azoles, and high mortality rates demonstrate a significant unmet medical need for novel antifungal agents with activity against these *Candida* species.

Fosmanogepix (FMGX; PF-07842805, APX001, E1211) is the first member in the “gepix” class of antifungals, with a unique mechanism of action (MOA). FMGX is a prodrug that is rapidly converted *in vivo* by systemic phosphatases to the active moiety manogepix (MGX). MGX inhibits the conserved fungal glycosylphosphatidylinositol (GPI)-anchored wall transfer protein 1 (Gwt1) (13, 14). In yeasts, Gwt1 mediates cross-linking of cell wall mannoproteins to β-1,6-glucan. Inhibition of Gwt1 results in pleiotropic effects on the fungal cell including alterations in fungal adherence to surfaces, inhibition of biofilm and germ tube formation, and subsequently results in severe growth defects and yeast lethality (15). Broad-spectrum antifungal activity has been observed against pathogenic yeasts and molds, including activity against resistant strains (16–18).

Surveillance studies have demonstrated that MGX was the most mycologically active agent against over 400 diverse C. auris isolates, including strains which were multi-drug resistant (19, 20). Another study that focused on 200 C. auris strains collected between 2017 and 2020 in New York and New Jersey reported low MICs for MGX even against pan-resistant strains shown to be resistant to the 3 main classes of antifungal drugs (i.e., azoles, polyenes, and echinocandins) (21). When *in vitro* activity of MGX was evaluated against eight comparator agents against 122 wild type and non-wild type C. auris isolates, similar findings were reported, with MGX having the most potent antifungal activity against all C. auris strains (22). FMGX improved survival over anidulafungin, an echinocandin, in a disseminated C. auris infection model in immunocompromised mice (13). In a study assessing the efficacy of FMGX in reducing fungal burden and increasing survival of C. auris infected neutropenic mice, similar *in vivo* efficacy and improved survival was detected, even when treatment was delayed postinfection by 24 h (23). Additionally, with a wide tissue distribution, high oral bioavailability, and availability in both IV and oral formulations, FMGX provides drug characteristics that are favorable for treating invasive fungal infections, such as candidemia/invasive candidiasis caused by C. auris (24, 25).

In a Phase 2 study in 20 patients with invasive candidiasis, FMGX was safe, well-tolerated, and demonstrated a high treatment success rate of 80% at end of study treatment (EOST) and Day 30 survival of 85% (26). In the present Phase 2 study, we aimed to assess the efficacy and safety of FMGX in a similar population of patients with candidemia/invasive candidiasis caused by C. auris, with limited antifungal treatment options.

## RESULTS

### Disposition, demographics, and exposure.

The study enrolled 9 participants from 2 sites in South Africa (15 participants were planned for enrollment). However, the study was terminated early due to the impact of the COVID-19 pandemic on patient enrollment. The intent-to-treat and safety populations each included all 9 enrolled participants with candidemia (henceforth, study population). Eight participants completed a full course of treatment, and one did not complete treatment due to death. Seven participants completed the study through the 4-week follow-up, with an additional death reported during the follow-up period.

Demographics and baseline characteristics for enrolled participants were consistent with the epidemiology of C. auris infections (Table 1). The study was conducted in intensive care units (ICUs). Underlying diseases leading to C. auris infection included fractures, burns, trauma, and nervous system disorders. All participants had C. auris infections at baseline with no coinfections with other *Candida* spp. All participants had candidemia only, with no evidence of invasive candidiasis at other sites. All 9 patients received at least 1 dose of an echinocandin antifungal (for ≤4 days) prior to start of FMGX treatment. No other class of antifungal was administered prior to FMGX treatment.

**TABLE 1:** Demographics and baseline characteristics

Parameter	Fosmanogepix *N* = 9
Age, mean (SD^*a*^) [range] yrs^*b*^	49.8 (17.7) [21 to 76]
<65 yrs, *n* (%)	7 (77.8)
≥65 yrs, *n* (%)	2 (22.2)
Gender, *n* (%)	
Male	6 (66.7)
Female	3 (33.3)
Race, *n* (%)	
Black or African American	5 (55.6)
White	3 (33.3)
Asian	1 (11.1)
BMI^*c*^ (kg/m^2^), mean (SD)	28.11 (6.6)
APACHE^*d*^ II score, mean (SD)	12.7 (6.4)
<10	3 (33.3)
10 to 19	4 (44.4)
20 to 30	2 (22.2)
ICU^*e*^, *n* (%)	9 (100.0)

a SD, standard deviation.

b yrs, years.

c BMI, body mass index.

d APACHE, acute physiology and chronic health evaluation.

e ICU, intensive care unit.

The mean (SD) duration of treatment with FMGX was 19 (5.83) days, ranging from 11 to 27 days; most participants (5/9 [55.6%]) received FMGX for >14 days but ≤28 days. All participants received FMGX via IV infusion; none were switched to the oral formulation. PK sampling was sparse and relatively few PK samples were collected, precluding a model-based compartmental analysis. However, a linear, two-compartment population PK model-based on previous phase I studies provided an adequate fit for the MGX concentrations observed. The PK data collected indicated that all participants had systemic exposures to MGX (median [range] total drug plasma AUC over duration of therapy: 93 [64.4 to 182] mg·h/L), consistent with prior exposure data from Phase 1 and Phase 1b studies in healthy volunteers and in patients with acute myeloid leukemia. MGX AUC:MIC ratios were also determined (median [range] total drug plasma AUC:MIC ratio over duration of therapy: 7147 [5194 to 12112] mg · h/L). Attainment of the total-drug plasma AUC:MIC ratio ED_50_ target was 100% for total-drug plasma AUC:MIC ratio evaluated on Day 7 and averaged over study treatment. FMGX exposures were minimal, as expected, given the rapid conversion by systemic phosphatases of parent (FMGX) to active moiety (MGX), as observed previously in Phase 1 and Phase 1b studies.

### Efficacy.

**(i) Primary efficacy endpoint: Treatment success at EOST.** DRC-assessed treatment success at EOST was 89% (8/9) in the study population (Table 2). One (11%) participant was a treatment failure at EOST as death occurred during the FMGX treatment period. The participant initially presented with extensive burns (60% partial to full thickness) and acute renal failure 27 days prior to enrollment. The participant eventually developed Gram-negative sepsis and multi-organ failure on Day 10 of the study and died due to cardiac arrest on Day 11. This death was considered unrelated to FMGX. All 4 patients who did not have all preexisting intravascular catheters removed prior to receiving the first dose of FMGX were assessed as treatment successes by the DRC, as were the majority (4/5) of the patients who had their intravascular catheters removed.

**TABLE 2:** Efficacy endpoints: treatment success and survival

Endpoint	Fosmanogepix *N* = 9
Response at EOST^*a*^	
Treatment Success^*b*^, *n* (%) [95% CI^*c*^]^*d*^	8 (88.9) [51.8, 99.7]
Treatment Failure, *n* (%)	1 (11.1)
Reasons for failure at EOST	
Death, *n* (%)	1 (11.1)
Response at 2 wks after EOST	
Treatment Success Sustained, *n* (%) [95% CI]^*d*^	6 (66.7) [29.9, 92.5]
Clinical Relapse (Positive culture), *n* (%)	1 (11.1)
Death, *n* (%)	1 (11.1)
Response at 4 wks after EOST	
Treatment Success Sustained, *n* (%)	6 (66.7)
Survival at Day 30, *n* (%)	8 (88.9)

a EOST, end of study treatment.

b Treatment Success was defined as meeting all of the following criteria: 2 consecutive blood cultures negative for *Candida* spp. and/or for participants with a deep-seated site of infection, at least 1 negative tissue culture or aspirate/fluid culture; alive at EOST; and no concomitant use of any other systemic antifungals through EOST.

c CI, confidence interval.

d 95% CIs were two-sided exact binomial CIs.

**Secondary efficacy endpoints. (i) Time to first negative blood culture.** All participants had positive C. auris blood cultures during the enrollment period. A positive blood culture was reported for 3 participants on Day 1 before starting FMGX treatment (*n* = 3). For these participants, the mean (SD) time to first negative blood culture was 8.7 (5.51) days.

In the study population, 6 (66.7%) participants demonstrated eradication of C. auris bloodstream infection at EOST, 2 were indeterminate due to missing blood cultures, 1 participant had a recurrence 2 days after stopping FMGX (based on a single positive blood culture but was asymptomatic) (Table 3). All subsequent blood cultures were negative and therefore later assessed as eradication by the investigator. There were no further recurrences during the 4-week follow-up period.

**TABLE 3:** Mycological outcomes

Mycological outcome	Total (*N* = 9)	no. of Participants by Treatment Outcome	DRC assessed treatment outcome
EOST			
Eradication	6	6	Treatment Success
Indeterminate	2	1: no EOST culture taken	Treatment Success
		1: death on Day 11	Treatment Failure
Recurrence	1^*a*^	1^*a*^	EOST Treatment Success, followed by early relapse
Follow-up period (2 and 4 wks after EOST combined)			
Eradication	7	1^*a*^	Recurrence at EOST, subsequently eradication (early relapse)
		6	Treatment Success sustained
Indeterminate	2	1 death on Day 11	Treatment Failure at EOST
		1 death on Day 36	Treatment Success at EOST, not sustained through follow-up
Recurrence	0		

a Patient with investigator-assessed mycological recurrence at EOST visit was assessed as treatment success followed by early relapse by DRC, because the single C. auris in blood culture was sampled 2 days after stopping study drug, *de facto* in the early follow-up period. All subsequent blood cultures were negative and assessed as eradication by the investigator. EOST, end of study treatment.

**(ii) Mycology.** All screened C. auris isolates recovered from blood cultures had low MIC values to MGX using both CLSI and EUCAST assay methods (0.008 to 0.015 μg/mL [CLSI] and 0.004 to 0.03 μg/mL [EUCAST]; Table 4). Among all antifungals evaluated, MGX had the lowest MIC values across all C. auris isolates. In the single case of relapse, the C. auris isolate did not have a high MIC value at baseline, nor did the value increase during FMGX treatment. For the study population, no shift in MGX susceptibility was observed in C. auris isolates collected during FMGX treatment. Similar to a previous study on C. auris isolates (22), the agreement between CLSI and EUCAST MICs for MGX were high (equal to or within 1 dilution for 8/9 isolates) while anidulafungin MICs were different (2 to 16-fold) between CLSI and EUCAST methods for all 9 isolates.

**TABLE 4:** Activity of manogepix and comparators against C. auris baseline isolates (CLSI and EUCAST methods)^*a*^

	MIC (CLSI^*b*^/EUCAST^*c*^; μg/mL)					
Participant	Amphotericin	Anidulafungin	Micafungin	Fluconazole	Manogepix	Voriconazole
1	1/0.5	0.5/1	0.25/0.25	>128/>128	0.015/0.015	2/2
2	1/0.5	0.5/0.06	0.12/0.03	>128/128	0.015/0.008	2/0.5
3	1/0.5	1/2	0.25/2	128/>128	0.015/0.03	2/8
4	1/0.5	1/0.25	0.25/0.25	>128/128	0.015/0.004	2/0.5
5	1/0.5	1/0.06	0.25/0.12	>128/>128	0.008/0.008	1/1
6	1/0.5	1/0.06	0.25/0.5	>128/>128	0.015/0.008	2/1
7	1/0.5	0.5/0.12	0.25/0.25	>128/>128	0.015/0.008	2/1
8	1/0.5	0.5/0.03	0.25/0.06	>128/>128	0.008/0.004	2/4
9	1/0.5	1/0.06	0.25/0.12	>128/>128	0.015/0.008	2/1

a MICs of isolates collected at baseline shown for all participants.

b CLSI, Clinical & Laboratory Standards Institute.

c EUCAST, European Committee on Antimicrobial Susceptibility Testing.

**(iii) Secondary safety endpoints.** All participants experienced a treatment-emergent adverse event (TEAE); however, none were considered treatment related (Table 5). The most common TEAEs were pyrexia (3 [33.3%]), constipation, multiple organ dysfunction syndrome, pneumonia, pruritus, hypertension, and hypotension (2 [22.2%] each). Two participants experienced 5 serious adverse events (SAEs); all were considered unrelated to treatment. No clinically significant safety concerns related to routine laboratory investigations were identified.

**TABLE 5:** Treatment-emergent adverse events^*a*^

Category	Fosmanogepix (*N* = 9)	
	Participants *n* (%)	Events *n*
All TEAEs	9 (100.0)	48
Study drug-related TEAEs	0	0
TEAEs^*b*^ by Maximum Severity		
Mild (CTCAE Grade 1)	1 (11.1)	2
Moderate (CTCAE Grade 2)	2 (22.2)	5
Severe (CTCAE Grade 3)	4 (44.4)	5
Life-threatening (CTCAE Grade 4)	0 (0.0)	0
Death (CTCAE Grade 5)	2 (22.2)	2
Study drug related TEAEs	0 (0.0)	0
SAEs		
All SAEs	2 (22.2)	5
Study drug related TESAEs	0 (0.0)	0
TEAEs leading to discontinuation of study drug	1 (11.1)	1
Study drug related TEAEs	0 (0.0)	0

a AE = adverse event; CTCAE: Common Terminology Criteria for Adverse Events; SAE = serious adverse event; TEAE = treatment-emergent adverse event; TESAE = treatment-emergent serious adverse event.

b TEAEs were defined as AEs occurring on or after the first dose of the study drug. Both participants and events are by maximum severity per patient.

Two deaths, both unrelated to FMGX treatment, were reported during the study. One occurred on treatment (Day 11) due to a cardiac arrest in a patient with Gram-negative sepsis, and the second occurred after treatment completion (Day 36) in a patient who developed ventilator-associated pneumonia and multi-organ failure.

## DISCUSSION

The goal of this Phase 2 study was to assess the safety and efficacy of FMGX in participants with C. auris candidemia and/or invasive candidiasis. The enrolled population was consistent with the disease epidemiology. All participants were in the ICU and were generally younger (median age of 45 years) and with fewer comorbidities than participants evaluated in prior candidemia and invasive candidiasis studies (27). A high rate of treatment success at EOST and survival at Day 30 (both 88.9%) was reported, along with a durable mycological response. No discontinuations or treatment related AEs were reported. Furthermore, both deaths were considered unrelated to study treatment. MGX was the most active compound tested against all C. auris study isolates with an MIC range of 0.008 to 0.015 μg/mL (CLSI) and 0.004 to 0.03 μg/mL (EUCAST). This is consistent with previous *in vitro* studies showing MGX to be the most potent antifungal tested against C. auris isolates (21, 22).

C. auris, a well-documented global threat, has caused several independent outbreaks in different geographical areas. Currently, 5 clades have been identified, based on genetic sequence and geographical origin (2, 7). Outbreaks with high fatality have occurred in health care settings in immunosuppressed patients. More importantly, rapid nosocomial spread from person-to-person has also been documented (28). Almost all identified strains are resistant to at least 2 classes of antifungals (azoles and polyenes), with some strains demonstrating multi-drug resistance (azole-polyene-candin) (29). Given these characteristics, C. auris is classified as an urgent global threat by the Centers for Disease Control and Prevention (CDC), with endemic hospital-associated infections being reported worldwide (30). There is a high unmet need for novel antifungals to treat candidemia/invasive candidiasis caused by drug resistant C. auris.

In the United States, the occurrence of infection caused by pan-resistant C. auris isolates was recently reported in several regions. These isolates were resistant to echinocandins, the antifungal class of choice for the treatment of most systemic candida infections (31). It is important to note that these patients all had prior epidemiological links to health care facilities but no prior echinocandin exposure (32).

In addition, outbreaks of C. auris infection are associated with increased length of hospital stay and health care costs, treatment failures, and death. In an analysis of an outbreak involving 34 patients at a large teaching hospital in the United Kingdom, prior antifungal prescription was strongly associated with increased risk of infection. In this case, the cost of infection control was estimated to be more than £1 million, notwithstanding costs related to lost opportunity, consumables, and screening costs. At the same health care facility, outbreak management costs continued to remain high the subsequent year due to an increased persistence of C. auris in the environment (33).

The incidence of C. auris infections have also increased during the COVID-19 pandemic, straining health care capacity, and increasing the risk of multidrug resistant nosocomial infections (34). Thus, there is an urgent need to improve treatment options for affected patients and control the spread of infection in health care facilities worldwide. Although no patients in this study received oral treatment, FMGX has high oral bioavailability and maintenance of plasma exposures when switching from IV or oral dosing. In addition, FMGX has a wide tissue distribution and provides exposures to the eye, gut, and brain, common sites of *Candida* dissemination (24).

This pilot study had some limitations. The sample size was small since the study was terminated early due to the ICU burden of the COVID-19 pandemic. However, no trial related visits or procedures were negatively impacted by the pandemic, and all protocol mandated study data were collected. All active sites from which participants were enrolled were in South Africa, and the C. auris isolates were likely from Clade III so these results may not be generalizable to all clades. However, Clade III isolates (found in South Africa) have also been detected in other countries, including Canada and the United States (2, 35). In addition, study participants were at high risk of infection with C. auris. A high rate of treatment success at EOST was achieved despite an increased risk of adverse outcomes in participants admitted to intensive care units. However, although none of the study participants had deep-seated C. auris infections, in a delayed therapy animal infection model of C. auris invasive candidiasis, significant reductions in fungal burden were observed in both kidney and brain (consistent with a deep-seated infection) in all dosing cohorts of FMGX in the survival arm of the study (23).

In conclusion, FMGX was safe and well-tolerated and demonstrated activity in participants with candidemia caused by C. auris. Based on the results from this pilot study, as well as a previously completed Phase 2 study in patients with candidemia, FMGX has the potential to be a safe and effective treatment option for patients with candidemia/invasive candidiasis. Additional studies are planned to further assess the safety and efficacy of FMGX in patients with invasive fungal infections.

## MATERIALS AND METHODS

This multicenter, open-label, non-comparative, single-arm study (NCT04148287), evaluated the safety and efficacy of FMGX for the treatment of candidemia and/or invasive candidiasis caused by C. auris. The study was planned at 4 sites in South Africa and one site in Panama. Participants were enrolled at 2 sites in South Africa, between 10 Dec 2019 and 23 Oct 2020, in accordance with International Conference on Harmonization Guidelines for Good Clinical Practice, applicable regulatory requirements, and the Declaration of Helsinki.

Eligible participants (males and females, ≥ 18 years of age) had an established diagnosis of candidemia and/or invasive candidiasis caused by C. auris from blood collected <120 h (for participants with candidemia) or from a normally sterile site <168 h (for participants with invasive candidiasis without candidemia) from the time of enrollment, and had limited or no treatment options due to either antifungal resistance, contraindication(s), intolerance, or lack of clinical response to standard of care antifungal therapy, as advocated by the relevant regional/country treatment guidelines (36, 37).

Mycological and clinical diagnoses criteria were: ≥ 1 positive culture from blood or other normally sterile site for C. auris (including possible co-infection with other *Candida* spp., except C. krusei), attributable clinical signs (fever [>38°C], or hypothermia [<36°C], or hypotension [SBP <90 mmHg or decrease of >30 mmHg]) within the permitted 120 h or 168 h enrollment period for candidemia or invasive candidiasis, respectively.

Exclusion criteria included severe or moderate hepatic impairment (total bilirubin > 3x upper limit of normal and alanine aminotransferase or aspartate aminotransferase > 5x upper limit of normal); concomitant use of strong CYP inhibitors; investigator-assessed life expectancy <7 days; diagnosis of C. krusei infection, deep-seated *Candida*-related infections requiring >42 days of treatment; and pregnancy or lactation. Participants with severe or moderate renal impairment were initially excluded, but became eligible later on, based on a protocol amendment.

### Treatment.

**FIG 1:**
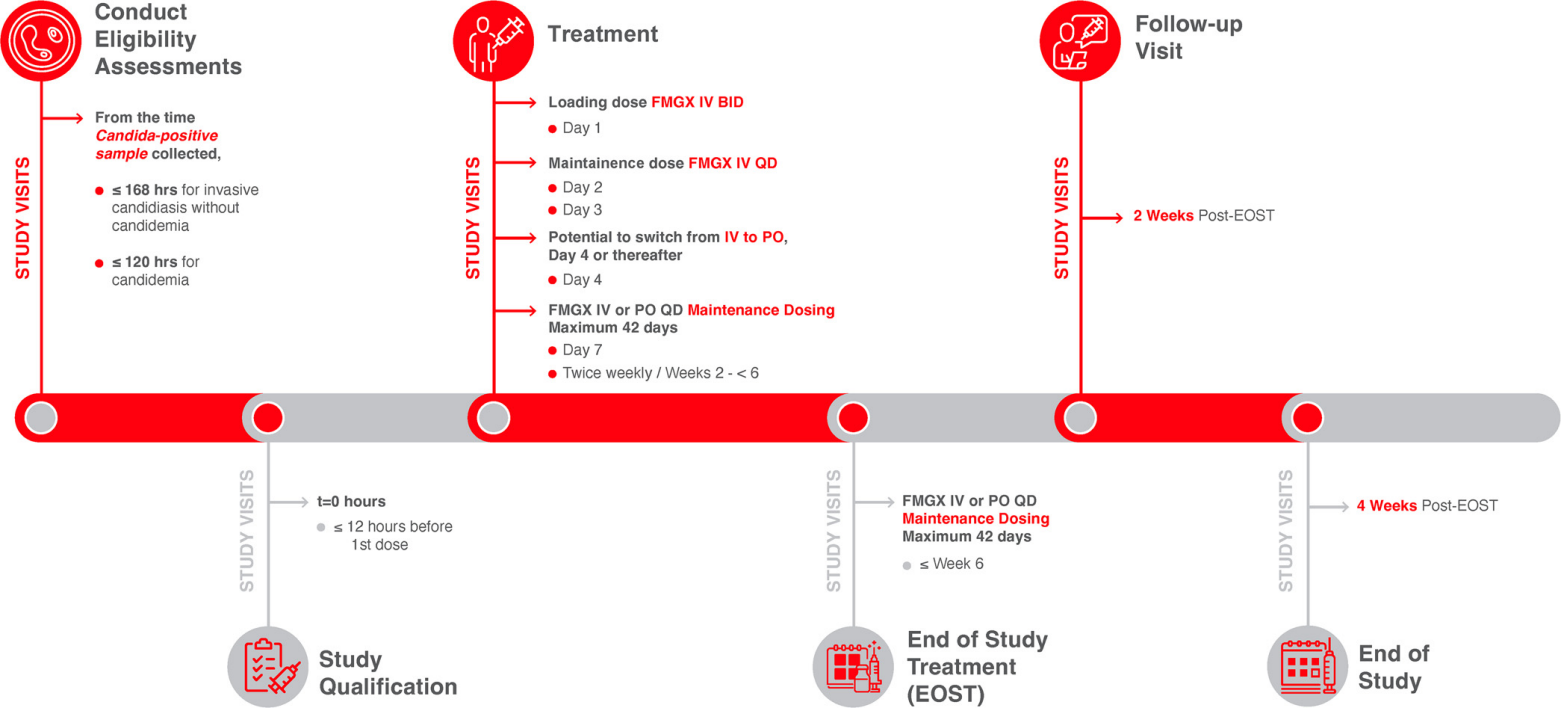
Study Design. For participants with invasive candidiasis, maintenance dosing was to continue until there was at least 1 negative tissue culture or aspirate/fluid. For participants with a deep-seated site of infection from which a tissue culture was not obtainable, maintenance dosing was to continue until after the resolution of the attributable clinical signs of infection recorded at baseline, and as applicable, radiological improvement associated with the site of infection. BID, twice daily; EOST, end of study treatment; FMGX, fosmanogepix; IV, intravenous; PO, orally; QD, once daily.

Participants were treated with FMGX within 12 h of study enrollment (Fig. 1). Participants received a 1000 mg FMGX loading dose (3-h IV infusion, twice daily [BID], 12 h apart). The maintenance dose was 600 mg IV, administered as a 3-h infusion once daily (QD). A switch to oral FMGX on Day 4 onwards (800 mg QD maintenance dose) was permitted at the discretion of the investigator if the participant was clinically stable and able to swallow tablets. FMGX was administered for 14 days after clearance of *Candida* from the bloodstream (2 consecutive negative blood cultures), and in accordance with clinical judgment as applicable for other infected sites, up to a maximum of 42 days. Participants were monitored for continued safety and efficacy at 2 weeks and 4 weeks after EOST.

Plasma samples for pharmacokinetic (PK) analysis of FMGX and MGX levels were collected at baseline (pre-dose), twice weekly during treatment, at EOST/early termination (ET), and 2 weeks after EOST. Adverse events (AEs) were recorded from the date of informed consent through study completion, and coded (using Medical Dictionary for Regulatory Activities [MedDRA] version 22.0).

The primary efficacy endpoint was the percentage of participants with DRC-assessed treatment success at EOST, defined as clearance of C. auris from the blood/infection site with no additional antifungal treatment and survival. Clearance was defined as 2 consecutive blood cultures negative for *Candida* spp., and/or ≥ 1 negative tissue culture or aspirate/fluid culture for deep-seated infections. For participants with a deep-seated infection involving visceral organs from which a tissue culture was not obtainable, resolution of the attributable clinical signs of infection that were recorded at baseline, and as applicable, radiological improvement associated with the site of infection, were required.

Secondary efficacy parameters included all-cause mortality through Day 30, and time to first negative blood culture. The DRC also assessed treatment success at 2 and 4 weeks after EOST. Investigator-assessed secondary parameters included percentage of participants with successful mycological outcomes, treatment success at EOST and 2 and 4 weeks after EOST, and the number of participants with TEAEs.

### Data availability.

Upon request, and subject to review, Pfizer will provide the data that support the findings of this study. Subject to certain criteria, conditions and exceptions, Pfizer may also provide access to the related individual de-identified participant data. See https://www.pfizer.com/science/clinical-trials/trial-data-and-results for more information.
